# Transactions of the New-York State Medical Society, February 1845

**Published:** 1845-07-01

**Authors:** 


*Transactions of the New-York State Medical Society, February* 1845. —These make a pamphlet of 177 pages, 82 of which are devoted to the annual address of the President, together with several communications on topics connected with medicine and the medical Profession; and the remainder to the doings of the Society, &c.

We know not what provisions are usually made to distribute copies of the annual transactions; but we fear that hitherto their circulation has been quite limited, even among members of the Profession in this state. It ought not so to be. They possess intrinsic interest and importance enough, to render it very desirable that the Profession generally in this state, and elsewhere, should have access to them; and if they fail to represent fairly the present condition of medical Science and the medical Profession in the Empire state, this will be the most effectually attained by giving to them as much publicity as possible, thereby enhancing the personal interest and responsibility of the members of the Profession in their character. We are glad to see among the resolutions of the Society, one recommending to the several county Societies to furnish each member with a copy. We hope the Societies will consider this recommendation, and act accordingly.

The address of the President, Joel A. Wing, M. D., is upon the Subject of *Spinal Irritation.* His remarks are wholly of a practical character, and appear to be based on his own experience. They coincide, mainly, with the observations of others who have devoted attention to this interesting and important department of Practical medicine. We commend the article to the perusal of our readers, and especially to those (if there are any such) who are still neglectful of the subject. By far the larger proportion of cases which fall under the observation of the practitioner, according to our experience, involve to a greater or less extent, the pathological relations of the spinal cord, and the Physician who fails to recognize these relations, must constantly find himself in a labyrinth of symptoms, without any clue to guide his steps.

The succeeding papers are communications transmitted from County medical Societies, and are as follows: *on the condition of the medical Profession, by G. W. Green, M. D.*; *on the progress of medical Science, by Simon A. Cook, M. D.*; *case of Lithotomy, in the female, by A. Baker, Jr., M. D; Medical Education and Legislation, by Geo. H. White, M. D.; Botany, by Dr. Joseph Bates.* They are all creditable productions, but our limits preclude any analysis of them, and we must therefore refer the reader to the articles themselves.

A large share of the proceedings of the Society relate to Medical Legislation and Medical Reform. With reference to these important, and, at the present time, exciting topics, several communications were received from various county Societies. The predominant sentiment appears to be against the propriety of petitions or applications of any nature whatsoever to the state Legislature. In this view we heartily concur. Of the prominent plans which are proposed by those who desire to see a change in existing laws regulating the practice of Physic and Surgery, and in general, of the subjects of medical Legislation, Education, &c., we purpose to offer some remarks hereafter. But without reference to the abstract merits of these subjects, we think that at the present juncture, a proper sense of self-respect requires that the matter should be left entirely in the hands of Legislators and the Public So long as it is imagined that medical Science, and the medical Profession, are dependent on Legislative protection for their character, and capability of usefulness; while our uncompromising position with regard to Quackery in its Protean shapes is attributed to motives of selfish jealousy or apprehension, instead of a philanthropic regard for the safety and welfare of the Public— in short, until a more just appreciation of medicine, as a science and profession, more generally obtains, we are clearly of opinion that a due regard alike for our own dignity, and the public weal, forbids any effort, on our part, to obtain the enactment of new laws, on any modifications of those which now exist. We have no fears but that the time will come, when the Public will become satisfied that its own interests are chiefly involved in the issue which has been made between regular and irregular practitioners; and that the time will come too when the co-operation of the profession for the public security and welfare will be as anxiously solicited, as it is now repelled with mistrust and insult. To persist in volunteering our aid, unasked and undesired, would not only be derogatory and useless, but would tend by sustaining prejudice to defeat the very object which is held in view. As to the question whether the Profession would not do better independently of all Legislation, we will not in this connection advance an opinion, but we may discuss it at some future time. The action of the Society was apparently in accordance with the preceding views, since the whole matter was postponed until the next annual meeting.

We should be glad to give with some detail an analysis of the various reports, &c., which came before the Society, if our limits permitted. This would be hardly desirable, however, if the work itself is accessible to our readers, which we hope will be the case. The proceedings of the annual meetings of the state Society should be looked for with interest by the Profession. At the present time, especially, they are to be regarded as particularly important. The dignity, usefulness and elevation of the profession, are committed to its own hands. To promote these objects, its members must act in concert; and this can only be attained by a body, composed of delegates from the several county Societies, representing the opinions and feelings of the profession in different portions of the state. As a basis for proper deliberative and concerted action, the meetings of the County Societies should be fully attended, and the important questions of reform, education, &c, fully discussed. The delegates to the state Society should be carefully selected, and they should feel it incumbent upon themselves to attend, unless prevented by extraordinary circumstances. In this way a full attendance at the annual meetings of the state Society would be secured, and delegates would go qualified to represent their constituents, and prepared to act in their behalf. On the whole, we have read the proceedings of the last meeting of the Society with gratification. They are characterized by moderation, dignity and good taste. We are not ashamed to have the published transactions circulate abroad as evidences of the ability and spirit which pervade the profession in the Empire State. Still there is room for improvement; and if a more general emulation could be aroused among the members of the county Societies to contribute papers with a view to their communication to the state Society, we believe there might be collected an amount of valuable material, selections, from which, would be in an eminent degree useful, and confer honor on the Society and State. In this remark we mean no disparagement to the communications which have hitherto appeared. We have already testified to the very creditable character of those which appear in the transactions of the present year, and we doubt not that their authors would freely admit the correctness of the opinion which we have advanced

